# In Vivo Delivery of Nucleic Acid-Encoded Monoclonal Antibodies

**DOI:** 10.1007/s40259-020-00412-3

**Published:** 2020-03-10

**Authors:** Ami Patel, Mamadou A. Bah, David B. Weiner

**Affiliations:** Vaccine and Immunotherapy Center, The Wistar Institute, 3601 Spruce Street, Philadelphia, PA 19104 USA

## Abstract

Antibody immunotherapy is revolutionizing modern medicine. The field has advanced dramatically over the past 40 years, driven in part by major advances in isolation and manufacturing technologies that have brought these important biologics to the forefront of modern medicine. However, the global uptake of monoclonal antibody (mAb) biologics is impeded by biophysical and biochemical liabilities, production limitations, the need for cold-chain storage and transport, as well as high costs of manufacturing and distribution. Some of these hurdles may be overcome through transient in vivo gene delivery platforms, such as non-viral synthetic plasmid DNA and messenger RNA vectors that are engineered to encode optimized mAb genes. These approaches turn the body into a biological factory for antibody production, eliminating many of the steps involved in bioprocesses and providing several other significant advantages, and differ from traditional gene therapy (permanent delivery) approaches. In this review, we focus on nucleic acid delivery of antibody employing synthetic plasmid DNA vector platforms, and RNA delivery, these being important approaches that are advancing simple, rapid, in vivo expression and having an impact in animal models of infectious diseases and cancer, among others.

## Key Points

**Table:** 

Direct in vivo delivery of synthetic nucleic acid-encoded antibodies employing plasmid DNA [plasmid DNA-encoded monoclonal antibodies (pDNA-mAbs)] and messenger RNA-encoded monoclonal antibodies (mRNA-mAbs) platforms represent new approaches for the in vivo delivery of antibody-like biologics.
While there are more preclinical data using pDNA-mAbs, both platforms have made significant progress and are demonstrating promising efficacy in infectious disease and cancer studies in small and large animal models.
These platforms have advantages such as rapid product development and simpler manufacturing processes, yet they represent different strategies for deployment, with unique advantages and challenges.

## Antibody Therapy

**Fig. 1:**
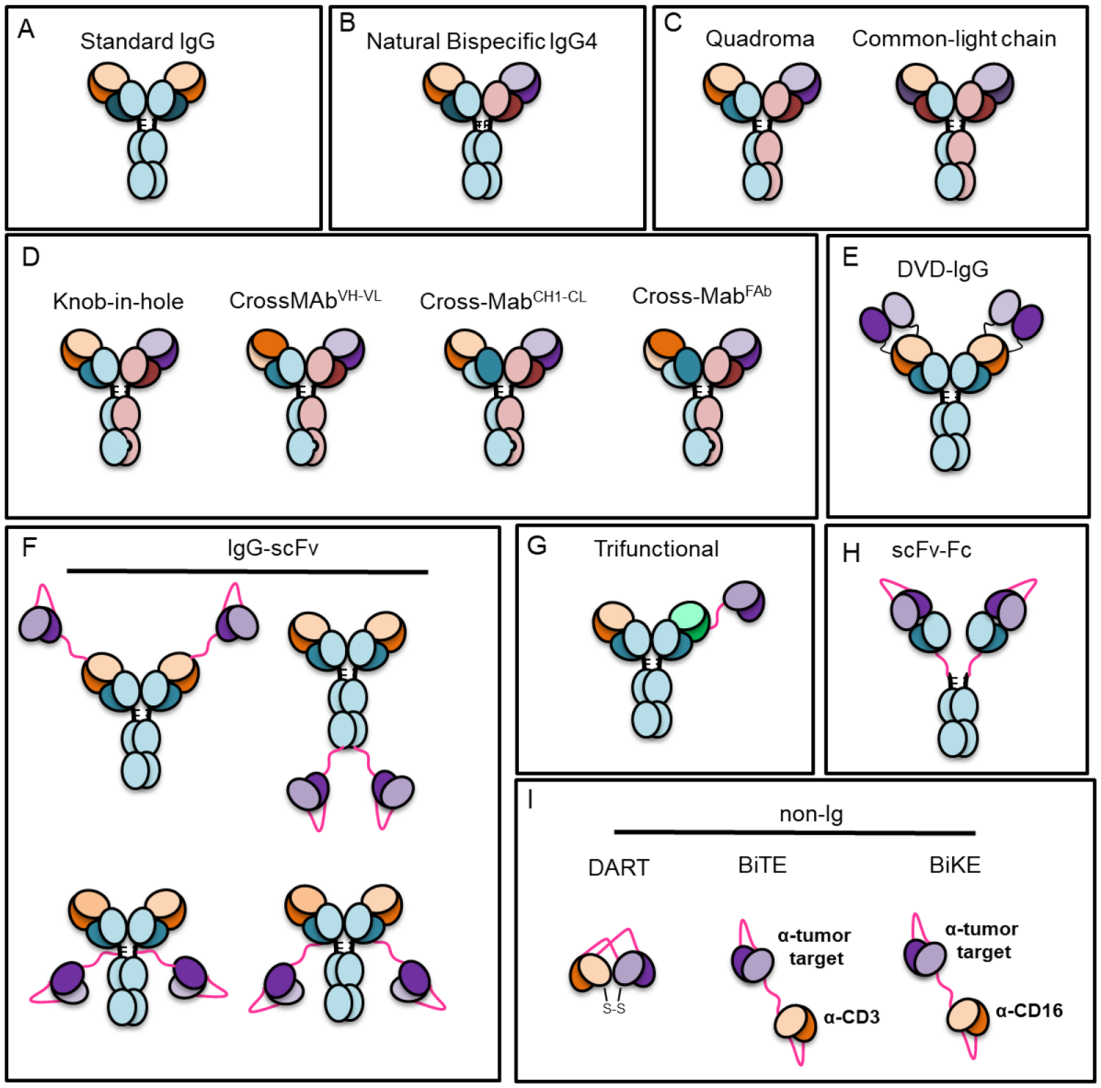
Different types of traditional Ig, bispecific Ig, and non-Ig designs. *Ig* immunoglobulin, *BiTE* bispecific T cell engager, *BiKE* bispecific natural killer engager, *scFv* single-chain variable fragment, *DART* dual affinity retargeting, *DVD-IgG* dual variable domain immunoglobulin

Monoclonal antibody (mAb) therapy has changed the landscape of modern medicine. To date, there are over 80 different mAb biologics approved by the Food and Drug Administration (FDA) and European Medicines Agency (EMA) for treatment of infectious diseases, cancer, asthma, and autoimmunity, among others. With these successes, the field is expanding into new and exciting related areas for biologics that target multiple specificities. This includes a range of bispecific and trispecific mAbs which can bind to the same or multiple antigens. Among the newest mAb-related biologics are bispecific T-cell engagers (BiTEs), bispecific and trispecific killer cell engagers (BiKEs and TRiKEs), and dual-affinity re-targeting antibodies (DARTs), as well as many others (reviewed in Ref. [1] and Fig. 1). These developments are the direct result of over 40 years of continuous advances in mAb isolation approaches, including hybridoma technologies, yeast surface display, phage display, and, more recently, single B-cell sorting strategies that identify paired heavy chain and light chain from single cells, among others (reviewed in Refs. [2] and [3]). New structural engineering strategies to improve potency and cell culture production, as well as the parallel development of sophisticated bioprocess manufacturing technologies, have additionally contributed to meet the growing demands for mAb biologics [4].

**Fig. 2:**
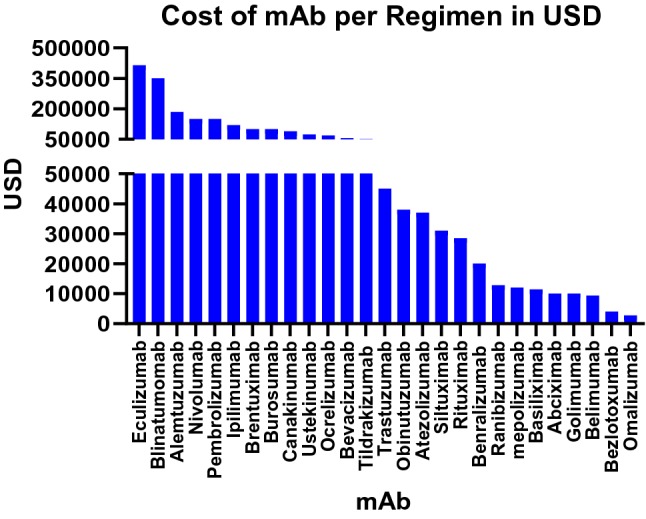
Cost of mAb per regimen (USD): the cost in USD for a single regimen of several mAbs based on 2019 first quarter Average Sale Price Files set by U.S. Centers for Medicare and Medicaid Services. *USD* U.S. dollars

Despite these many advances, large-scale bioprocessing is faced with challenges that currently hamper wider global deployment. The intrinsic biochemical and biophysical properties of antibody sequences are frequent liabilities for large-scale manufacturing and may also lead to post-manufacturing aggregation and stability issues. Such limitations may prevent an otherwise highly potent and effective mAb from advancing through development and into the clinic [5, 6]. Delivery challenges must also be overcome as in vivo administration of mAb biologics often requires high doses (grams of mAb) to achieve therapeutic efficacy, frequently at a high cost (Fig. 2). While the cost of raw materials is relatively inexpensive, bioprocess manufacturing and purification can be lengthy and costly (reviewed in Ref. [7]). The price of mAb biologics is driven by a combination of research and development costs, duration of treatment, patient market size, geographic location, private insurance coverage, and availability of biosimilars [8, 9]. mAbs requiring higher doses need to be administered through slow intravenous (IV) infusions to limit infusion reactions. IV delivery frequently requires hours of clinical monitoring and may involve post-infusion monitoring for allergic or anaphylactic reactions, further increasing the medical personnel required and costs of administration. Subcutaneous (SC) delivery has advantages for lower dose antibody delivery, including being less invasive and the possibility for self-administration in several indications, such as rheumatoid arthritis, primary immunodeficiencies, and multiple sclerosis [10, 11]. Drug autoinjectors have greatly improved the uptake and convenience of SC delivery, also regulating dosing. However, SC delivery is associated with pain related to injection volume and injection site reactions, and absorption is slow due to reliance on the lymphatic system [12] for biodistribution [12]. As a result, the mAb may be eliminated before reaching systemic circulation (reviewed in Ref. [13]). Nonetheless, the impact and importance of mAbs on human disease and the growth in new applications of such technology cannot be understated.

With all mAb biologics, cold-chain storage and transportation is required to enable reasonable product shelf life. Temperature control is important for lengthening mAb biologic stability and storage. However, the need for a cold-chain can limit global distribution to at-need populations in resource-limited areas, and requires additional production runs to resupply patients, due to expiring product. For these reasons and others, novel creative approaches for in vivo gene-encoded mAb delivery building on existing biologic technologies are under investigation, with the goals of increasing patient accessibility and providing novel clinical options for this growing class of important biologics.

## In Vivo Antibody Gene Delivery

In vivo gene-encoded antibody delivery is an elegant approach that may address many limitations of traditional antibody biologics. In general, the antibody heavy- and light-chain complementary DNA (cDNA) are examined and then optimized or designed for encoding and expression specifically for the type of vector chosen for in vivo delivery. Historically, in the gene therapy arena, viral vectors [adeno-associated virus (AAV) is the current favored viral vector] have been promising for in vivo transfection and gene expression. More recently, the newly emerging non-viral synthetic nucleic acids [synthetic DNA formulated for facilitated delivery by electroporation, or formulated lipid nanoparticle (LNP) encapsulated messenger RNA (mRNA)] have started to receive considerable attention. The synthetic antibody is transcribed (in the case of AAV and synthetic DNA) and translated within the in vivo transfected targeted cells, followed by antibody secretion into the systemic circulation. RNA approaches use LNP to allow for delivery in vivo, and then the mRNA is directly loaded on ribosomes for translation in the cytoplasm, skipping the nuclear steps used by AAV and DNA deliveries. The synthetic nucleic acid field is rapidly providing insights into the in vivo biology of genetic expression which suggest novel exciting strategies for drug delivery, with broad implications for the prevention and treatment of disease. A high-level summary of synthetic DNA and synthetic mRNA and comparison with viral vector delivery platforms for in vivo mAb delivery is presented in Table 1.

**Table 1:** Comparison of different in vivo antibody gene delivery platforms

	Synthetic DNA	mRNA	Viral vector
Starting material	Plasmid DNA	Plasmid DNA	Plasmid DNA
Injected material	Plasmid DNA/by EP	mRNA in LNP	AAV, Ad vector
In vivo product	mAb, BsMAb	mAb, BsMAb	mAb, BsMAb
Transient	Yes	Yes	Yes; however, rare cases may integrate (AAV)
Cell compartment	Nucleus	Cytoplasm	Nucleus
Integration	None observed in humans	Low probability data early	Possible [14]
Anti-vector immunity	No	No	Yes, serotype specific
Duration of a single administration	Weeks/months	Days/weeks	Months/years
Delivery route	ID, IM	IV, IM/ID	IM, IN, IV, IC, IO, IT
Tissue specificity/tropism	Local site of administration	Non-specific	Serotype dependent
Vector take	High in animals	High in preclinical	Variable
Human safety	Very safe, no SAEs in 1000s of individuals	Ongoing trials, SAEs reported [15]	Safe but potential for integration (AAV)
Ease of manufacturing	Yes	Likely yes with caveats	Moderate
Cold-chain free	Yes	Possible (with LNP) No (without LNP)	No

*AAV* adenovirus-associated virus, *Ad* adenovirus, *BsMAb* bispecific mAb, *EP* electroporation, *IC* intracranial, *ID* intradermal, *IM* intramuscular, *IN* intranasal, *IO* intraocular (subretinal and intravitreal), *IT* intrathecal, *IV* intravenous, *LNP* lipid nanoparticle, *mAb* monoclonal antibody, *mRNA* messenger RNA, *SAEs* serious adverse events

### Viral Vector-Encoded Antibodies

The original studies surrounding in vivo antibody gene delivery focused primarily on gene delivery using recombinant viral vectors such as AAV and adenovirus (Ad), which were advanced clinically, building on work in the traditional gene therapy-based field. Among these choices, AAV has been the most studied option for mAb delivery, due to its particular advantages. These include the fact that it is more immune silent as a vector compared with other viral vector platforms and can achieve long persistence of the genetic cassette with a single administration (reviewed in Ref. [16]). Viral vector delivery is dependent on viral surface receptor-mediated entry into cells, with different vectors displaying unique tissue tropisms (e.g., liver, muscle, and lung) based on their surface capsid (AAV) [17] or hexon (Ad) [18] proteins. In vivo viral vectors encoding antibody genes have been reported to be administered either locally [via intramuscular (IM), intranasal (IN), intraocular (IO), or intracranial (IC) routes] or systemically via IV or intrathecal (IT) routes, leading to viral infection with production of the virus-encoded antibody predominately from liver and lung and perhaps other tissues (reviewed in Ref. [19]). Interestingly, AAV vectors take several weeks to reach their peak expression; therefore, to accelerate expression, De et al. evaluated a combination of both an Ad vector-encoded antibody followed by an AAV-encoded mAb targeting anthrax and demonstrated successful rapid and long-lasting expression [20]. Around the same time, Skaricic et al. demonstrated delivery of a murine version of the anti-respiratory syncytial virus antibody palivizumab using Ad serotype 5 and AAVrh.10 vectors, showing protection and expression for 20 weeks [21]. With additional vector promoter optimizations, AAV vectors are capable of high antibody expression, approaching 1 mg/mL serum concentration at the highest vector doses in mice [22]. High systemic AAV-mAb expression levels have been observed in macaques that have uptake of the transgene, typically in the range of up to 100 μg/mL in serum (reviewed in Ref. [23]) and as high as 270 μg/mL in a single macaque, with expression for 2 years. However, expression can decrease in animals when suppressed by host development of anti-vector cellular immunity or host anti-drug antibody (ADA) immune responses [24]. In humans, re-administration of AAV vectors can be difficult due to rapid development of serotype-specific antibodies.

AAV-mAbs have been studied for their ability to deliver anti-HIV broadly neutralizing antibodies. AAV serotypes 1, 2, and 8 have been evaluated as vector backbones for delivery of anti-HIV mAbs b12, VRC01, VRC07, and 10-1074, and immunoadhesins such as eCD4Ig (reviewed in Ref. [23]). However, it was observed that these viral vectors were limited to single-use delivery, as natural serology and vector-induced neutralizing antibodies impaired repeat administration. This reduces the potential applications for long-term, repeat delivery of anti-HIV AAV-mAbs or delivery of combinations of anti-HIV mAbs.

Development of additional AAV vector serotypes targeting different tissues and assessing different routes of delivery is ongoing. Many of the initial AAV-mAb studies were performed utilizing IM delivery. IV delivery is less targeted, resulting in viral vector delivery and production in multiple tissues, including liver, lung, and others. Limberis et al. have focused on AAV9 delivery to the lungs via IN delivery for anti-influenza virus [25–27], and Laursen et al. demonstrated AAV9 delivery of an anti-influenza multi-domain antibody [28]. Additionally, Limberis et al. described the delivery of anti-Ebolavirus AAV-mAbs [29]. However, a parallel study evaluating AAV9 delivery of the same anti-Ebolavirus antibodies by IM and IN delivery routes demonstrated different results based on the route of administration [30]. These data support the hypothesis that there may be variations in delivery take of AAV vectors [31] and that further studies investigating different routes of delivery continue to be important. Nevertheless, the data with viral vectors, particularly AAV-mAbs, are promising and supportive of the potential for an in vivo mAb delivery approach to administer highly potent antibodies. These vectors have unique advantages and disadvantages, supporting the need for more work in this area. Importantly, these studies serve as an important foundation for the development of additional in vivo mAb approaches using non-viral delivery platforms.

**Fig. 3:**
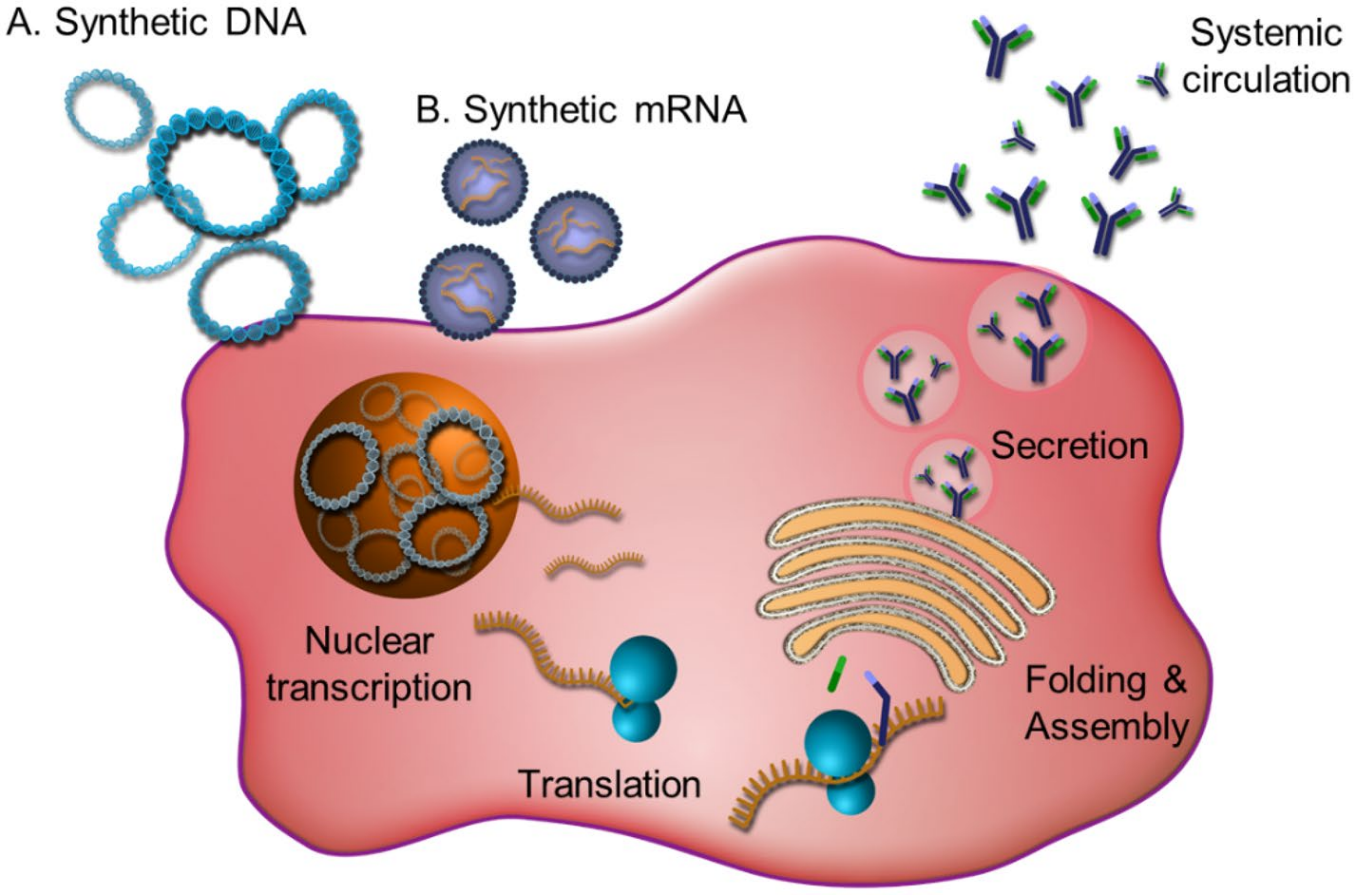
Nucleic acid antibody gene delivery using synthetic DNA or mRNA administration into the cell

We focus below more specifically on *non-viral antibody gene delivery* technologies using synthetic mRNA or synthetic plasmid DNA vectors (Fig. 3).

## Synthetic Nucleic Acid Delivery

DNA and RNA represent the nucleic acid building blocks of life. DNA must first be transcribed into mRNA before translation to proteins. In vitro-transcribed mRNA delivery circumvents in vivo transcription, leading directly to protein translation. Although related, there are significant differences between DNA- and mRNA-vectored antibody delivery approaches.

### Synthetic DNA

DNA is a highly stable, double-stranded regulated nucleic acid with antiparallel nitrogenous bases bonded by intervening deoxygenated pentameric sugar and a phosphate backbone capable of being read and faithfully transcribed into mRNA. This can generate logs of mRNA from a single DNA expression cassette. The naïve mRNA is then processed through enzymatic removal of noncoding introns and subsequent ligation of coding exons with the final addition of a 5′ 7-methyl guanosine and polyadenylated 3′ to buffer against nuclease activity, yielding a mature mRNA that is translated into protein by ribosomal enzymes [32, 33]. Initial DNA vectors were developed by direct cloning and expression of natural sequence inserts derived from the host. The recent change to synthetic generation of highly designed, synthetic inserts has had a major impact on the platform. Synthetic plasmid DNA vectors can be engineered to encode large, complex proteins. This is achieved through space-saving plasmid engineering encoding only designed coding sequences with enhanced leader and optimized stop sequence which are required for natural downstream mRNA processing and termination, but have improved performance compared to native sequences. The natural temperature stability of pure DNA means that synthetic plasmid DNA vectors can be stored at ambient temperatures for extended periods of time, as they can withstand significant temperature fluctuations without damage [34]. Synthetic DNA is therefore capable of being developed to be cold-chain independent, making it highly attractive for product development and administration in resource-limited settings. One dramatic difference compared with viral delivery is that synthetic DNA does not induce anti-vector backbone immune responses and a homologous synthetic DNA backbone encoding the same or a different gene can be re-administered over and over [35]. As a result, DNA is a promising platform for in vivo delivery of vaccines, protein replacement, or antibody genes, especially when combined with enhanced delivery, as discussed in Sects. 4, 5, 6, and 7. DNA is transiently expressed and can continue to produce protein as a depot until it is lost from cells. In general, as plasmid delivery is non-live, non-replicating, and non-integrating, these deliveries are transient.

Plasmid DNA-encoded mAbs (pDNA-mAbs) are engineered to carry synthetic antibody genes, similar to AAV-mAb platforms. The literature describes that pDNA-mAbs can exhibit peak serum concentrations after just 2 weeks and then can express at consistent levels for 2–3 months and decline thereafter as the plasmid is lost from cells and then cleared from the serum due to the natural half-life of immunoglobulin G (IgG) [36–41]. Long-term small animal studies show the total duration of pDNA-mAb expression to be at least 1 year [42, 43]. However, in contrast to viral vectors, synthetic DNA requires an efficient delivery system. By far the most advanced of these are the adaptive electroporation systems, where a consistency of in vivo DNA delivery and efficiency has been reported [44], representing potential utility for pDNA-mAb delivery, at least preclinically. In the clinic, vaccine delivery via facilitated electroporation has generated impressive data in people [45–47]. The delivery technology is discussed in Sect. 3.4.

### mRNA or Self-Amplifying (SAM) RNA

mRNA offers a platform with the ability to rapidly express protein, bypassing DNA to RNA processing and directly attaching to ribosomes in the cytoplasm for translation of the protein of interest. This results in a fast and efficient delivery, followed by a single round of expression leading to bolus protein expression and secretion. RNA, with its oxygenated 3′ sugar backbone, is inherently unstable, especially in alkaline pH, and prone to enzymatic activity and degradation. This increases the chances of RNA integration via reverse polymerase transcription processes. Synthetic mRNA must encode all the necessary RNA processing structures, including a 5′ cap in the correct orientation and a polyadenylation tail, limiting its overall coding capacity [48]. Frequent repeat administrations are required for long-term delivery, due to its short half-life [49].

Synthetic in vitro-transcribed (IVT) mRNA is more biochemically unstable compared to DNA, and this has driven the search for rapid parallel advances in various LNP formulations for packaging the RNA. LNPs improve overall stability as well as providing a means for IVT mRNA to enter target cells. Delivery by specific LNP has significantly reduced recognition of mRNA by innate sensors; however, mRNA must exit the LNP into the cytoplasm in order to be translated, and therefore still encounters sensors. This results in recognition by innate pattern recognition receptors such as toll-like receptors TLR3, TLR7, TLR8, and TLR9 that respond to mRNA by inducing inflammatory responses via endosomal compartments and retinoic acid-inducible gene-like receptors (RLRs), RIG-I, MDA5, and LGP2, that recognize unmethylated CpG nucleotides (or strands as in plasmid delivery) and single-stranded RNA to initiate degradation [50, 51]. RNA sensors can be particularly inflammatory. For this reason, modified nucleosides such as pseudouridine [52] and 5-methylcytidine are being advanced to enhance translation and stability, as well as lower innate inflammation via many of the innate immune sensors (reviewed in Ref. [48]). The limited studies of mRNA in the clinic have shown some adverse events at higher doses and are being watched closely, and work-arounds are in progress [53–55]. In contrast to mRNA, supercoiled plasmid DNA drives more limited innate stimulation of cytosolic DNA sensors (STING, c-GAS, and TBK1) [56–58] and activation of TLR9 [59], even in the context of advanced electroporation delivery. However, additional investigations and developments in this area are receiving significant attention to further improve mRNA delivery and prevent undesirable immune activation in dose escalation studies.

### Safety and Integration

Understanding the safety of gene delivery platforms is paramount for in vivo antibody gene delivery to progress. The safety data to date for both non-viral and viral vectors have been established from vaccine and gene therapy studies. Ongoing studies in humans with gene-encoded mAbs will be informative as the field moves forward (NCT03831503, NCT03829384).

Viral vectors have mostly been focused in the area of disease therapy, with few studies in normal healthy humans for gene therapy. In a limited number of studies, adverse events, including lymphopenia and neutropenia, among others, have been reported for Ad vectors [60], and concerns over genotoxicity are being raised for AAV (reviewed in Ref. [14]). This includes evidence suggesting genome integration by AAV vectors [61–64], potentially resulting in permanent rather than transient gene transfer. Such integration events are still unpredictable and require additional study to further elucidate. Higher doses of viral vector may circumvent pre-existing immunity; however, toxicity has been reported in dose escalation studies with Ad vectors [65, 66], including one fatality due to high-dose vector delivery [67, 68]. Similarly, high-dose AAV vector delivery in pig and non-human primate studies has also been associated with severe toxicity, under specific conditions [69], but most clinical studies which have focused on more moderate doses support that AAV delivery is very well-tolerated in the clinic. Excitingly, clinical impact has been achieved for specific applications [70], underscoring the importance of this approach. However, the utility of AAV for systemic delivery has continued to show challenges, with anti-vector immune responses, as well as breaking tolerance to the encoded transgene. This area is receiving additional attention. Additional development is important and is likely to eventually pay more dividends in the clinic.

The mRNA delivery field is still relatively new and there have only been a few mRNA gene therapy clinical trials and even fewer for delivery of antigens as vaccines or for in vivo encoded antibody delivery. The few mRNA studies reported have described primarily mild-to-moderate injections site reactions [55, 71]; however, these can have a high frequency, and occasionally they have been serious. In a small vaccination study, a serious adverse event was reported following administration of an mRNA-rabies vaccine [55]. The afflicted individual presented with transient Bell’s palsy, recovering after a second vaccination. Vaccine studies with an mRNA H7N9 vaccine demonstrated that intravenously delivered mRNA can disseminate to different tissues, such as the liver, heart, brain, kidney, and many others [54], with many individuals exhibiting less severe fevers and chills. The vaccine was immunogenic and produced serological responses in most vaccinated persons. The long-term impact of antibody production from many of these tissues is only beginning to be followed. As the field is still relatively new, additional studies and monitoring will be informative for the safety of mRNA delivery in people. As mRNA currently requires LNP formulation, further studies to evaluate the safety of different formulations in the clinic are ongoing. An ongoing important first-in-human trial to evaluate the safety of an anti-chikungunya virus (anti-CHIKV) mRNA-encoded mAb (mRNA-mAb) mRNA-1944 is recruiting (NCT03829384). The interim safety results from this study were reported at the Oligonucleotide Therapy Society Meeting (October 2019), with no adverse events reported in the lowest 0.1- and 0.3-mg/kg dose groups. However, grade 1, 2, and 3 adverse events were observed in the 0.6-mg/kg group [53]. Importantly, expression was observed of the encoded antibody, which is a very exciting development for the field. Microgram levels of antibody were reported after continuous administration of mRNA Ab for several hours by the IV administration route. These exciting results, while showing some challenges, are very important. Continued study and development in this area will provide important guideposts regarding the potency and the safety of this approach.

#### Understanding the Genomic Impact of mRNA and DNA Delivery

**Fig. 4:**
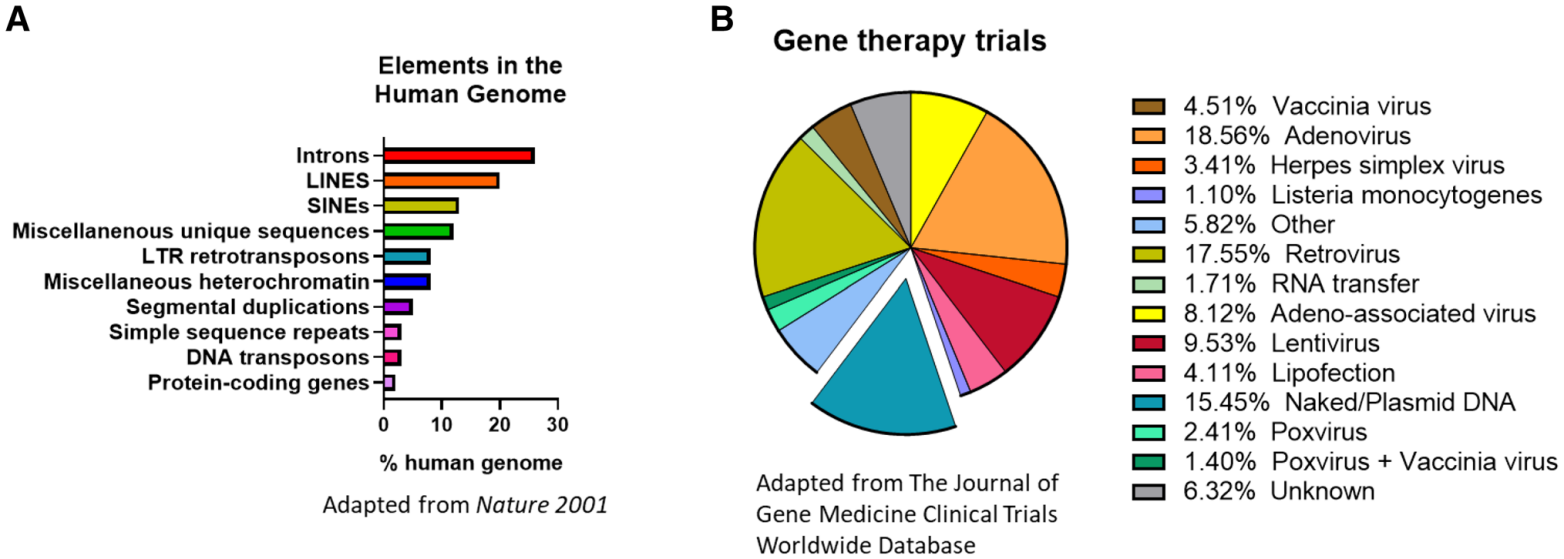
**a** Elements in the human genome (adapted from Ref. [73]), **b** breakdown of clinical trials by platform (adapted from Ref. [76]). *LTR* long terminal repeat

Further understanding of potential RNA methylation and the impact of epigenetic modifications is still needed. However, our understanding of RNA retrotransposons through long interspersed element (LINE) and short interspersed element (SINE) processes has informed much about the potential for DNA or RNA integration. The risk of integration for either platform is theoretical, which is not the case for many viral vectors. A survey of integration historical events in the human genome is illustrative. For example, specific elements such as LINEs and SINEs make up roughly 30% of the entire sequenced human genome and are distributed at intergenic and intragenic regions, respectively [72] (Fig. 4a) [72, 73]. Both LINEs and SINEs move through the genome via RNA intermediates that are converted to DNA before integrating into the genome at a different location. These transposons can have either an advantageous or deleterious effect. Findings by Kaessmann et al. and others have shown that, similar to SINEs, mRNA retrotransposons can also integrate with the help of LINE-encoded reverse transcriptase (RT) enzymes. These RT enzymes recognize polyadenylated mRNA destined to be copied into cDNA and lead to host integration of transposed genes with varying mutation and functional capacity [74]. Using retrotransposon capture sequencing (RC-seq), Baillie et al. were able to identify three active families of retrotransposons (L1, *ALU*, and SVA) that are known to have deleterious effects in humans by mobilizing nonspecific integration, which results in the abrogation of genetic functionality in somatic neuronal development [75]. Undoubtedly, these studies highlight the complexity and divergence of mRNA processes and the natural challenges and opportunities that exist to enhance a robust safety profile [73, 76].

Synthetic plasmid DNA has been extensively studied and has been administered to thousands of healthy human participants in numerous clinical trials over the past 3 decades. To date, there have been no reports of any serious adverse events related to plasmid DNA [77]. DNA delivery has a local biodistribution and stays at the site of initial injection, typically clearing within 2 months of administration [78]. The injection is well-tolerated, with only mild-to-moderate local (injection site) reactogenicity and no systemic toxicity observed in the thousands of persons studied, irrespective of higher dose administrations, in contrast to mRNA. Plasmid DNA delivery represents almost 15.4% of gene therapy vectors that have been investigated in human clinical trials (Fig. 4b) [76]. This number does not include the numerous human trials evaluating plasmid DNA vaccines against infectious diseases in normal healthy persons. The theoretical possibility for plasmid DNA integration is often cited as a risk factor for this platform. However, in experimental studies, plasmid integration events occur at rates *below* the statistical rate for DNA integration observed in nature [78], or the background mutation rate of normal exposure to the sun. To date, there have been no integration events in the preclinical studies in the tens of thousands of individuals receiving plasmid DNA, supporting the safety of synthetic DNA plasmid-based technologies like pDNA-mAbs. DNA transposons exist as part of < 3% of the genome; however, they are considered ancestral, and there is no evidence for new DNA transpositional activity for at least 37 million years [73, 79], again supporting the rare nature of such an event. Much of the activity of DNA transposons occurred during early primate evolution and appears to have ceased. Double-stranded DNA requires an additional integrase for genome insertion. This machinery is unlikely to be available in adult human cells. This is clearly unlike the LINE and SINE RNA intermediary retrotransposons that continue to exert mobile transpositional activity in the genome [80], suggesting additional study of the RNA in such events could be informative.

As nucleic acid-delivered antibody technologies begin to demonstrate interesting preclinical results, questions have arisen concerning how to control the magnitude and duration of in vivo expression. While long-lasting expression of antibodies targeting infectious pathogens is unlikely to be problematic, prolonged expression of checkpoint inhibitors could have lasting immune impact. Therefore, strategies that serve as on/off switches would be useful. Gene therapy studies with viral vectors have previously considered herpes simplex virus thymidine kinase (HSV-tk) as a potential “suicide” gene in combination with the antiviral ganciclovir. However, HSV-tk can be immunogenic [81] and is not active in non-dividing cells such as skeletal muscle [82, 83]. Alternatively, the inducible caspase-9 system (iCasp9) has also been studied. The iCasp9 system employs a modified caspase 9, combined with a homodimerization domain that is activated in combination with a chemical inducer of dimerization (CID), leading to apoptosis [84, 85]. Unlike HSV-tk, iCasp9 is active in non-dividing cells. iCasp9-based systems are being evaluated as safety switches for chimeric antigen receptor T-cell (CAR-T) engineering and therapy [86, 87]. In recent studies from the CAR-T field, Mestermann et al. employed the tyrosine kinase inhibitor dasatinib to suppress T-cell receptor signaling [88]. However, dasatinib has been shown to be toxic in skeletal muscle cells [89]. Furthermore, dasatinib does not completely remove CAR-T from circulation, rather it suppresses activity. In addition, the ligand-inducible RheoSwitch Therapeutic System has been evaluated in animal models [90] and people for regulation of interleukin-12 delivered by an Ad vector [91, 92]. Building on these studies from the gene therapy and CAR-T fields, additional study of gene safety switches in skeletal muscle would be useful for pDNA-mAb platforms.

Recently, the first phase I human trial evaluating the safety, tolerability, and pharmacokinetics of an anti-Zika virus DNA-encoded mAb (DMAb) (NCT03831503, estimated to complete in early 2021) has opened. This study will provide additional important data in regard to safety, the initial pharmacokinetics of particular formulations, and the delivery of DMAb in the clinic.

### In Vivo Synthetic DNA Delivery

Plasmid DNA is administered by a local injection. To date, this has been performed focusing on IM delivery strategies; however, other routes such as intradermal (ID) may be important as the platform advances. Early studies used needle and syringe delivery or older delivery devices, inducing very low in vivo expression levels [93, 94]. More recently, through engineering the inserts for increased in vivo expression and using more advanced systems like adaptive CELLECTRA™ electroporation, improved levels of in vivo antibody expression have been reported in many animal studies. The adaptive electroporation utilizes a direction-changing field, with depth-sensing novel technology and integrated resistance sensing. This greatly improves DNA delivery tolerability, as well as enhances in vivo transfection efficiency approximately 500- to 1000-fold, leading to a direct increase in protein expression [44]. pDNA-mAbs delivered using CELLECTRA™ technology are referred to as DMAbs. Other clinical systems, such as the Ichor TriGrid system [36] and the Igea Cliniporator (IGEA) [40], are also being employed for pDNA-mAb delivery, with demonstration of in vivo expression and impact in small animal studies.

Advancements such as hyaluronidase pre-treatment of muscles to transiently facilitate dispersion through the extracellular matrix (ECM), coupled with updated electroporation, have greatly enhanced this approach [95, 96]. In the early pDNA-mAb studies, Yamazaki et al. observed 4-ng/mL expression in mice before hyaluronidase pre-treatment and as high as > 15 μg/mL following hyaluronidase pre-treatment in mice [97]. Studies by several other groups displayed similar increases in in vivo expression following hyaluronidase pre-treatment [37, 39, 98]. A recent study by Schommer et al. utilizing chondroitinase ABC to degrade chondroitin, also present in the ECM, describes this enzyme as an alternative to hyaluronidase [99]. More recent studies have addressed the need for hyaluronidase pre-treatment and are utilizing co-formulation with recombinant human hyaluronidase (Hylenex^®^) in non-human primates [43]. The Zika DMAb phase I trial is utilizing this co-formulated approach.

### Plasmid Optimization

The most studied promoters for DNA-mAb delivery are associated with the use of the human cytomegalovirus (CMV) promoter [36–39, 42, 100–102] or the chicken beta-actin (CAG) promoter [41, 97]. Learning from the mRNA field, further optimizations in the 5′ and 3′ untranslated regions (UTRs), polyadenylation signals, and other post-translational response elements should also provide added benefits. Other strategies include evaluating heavy chain (HC) and light chain (LC) gene delivery on one versus two plasmids. In several early studies, it was demonstrated that two-plasmid delivery affords superior expression to single-plasmid delivery [38, 42]. Additional study is necessary in order to understand the biological reason for these expression differences and improve on strategies to develop single-plasmid pDNA-mAbs as well as other modifications. As the pDNA-mAb platforms advance, considerations such as shrinking plasmid backbones, including studies of closed linear DNA and minicircles, as well as linear deliveries represent interesting alternative options that are worth exploration.

### Sequence Optimizations

Analysis of antibody sequence liabilities and evaluation of “developability” criteria are essential for recombinant protein manufacturing [5, 6]. Interestingly, many of the rules that have been thoroughly studied over decades to establish traditional antibody manufacturing are unique with regard to in vivo mAb expression [5, 6]. Patel et al. made the first observation that in vitro pDNA-mAb expression data did not correlate with in vivo expression levels using the DMAb platform [42]. For example, recombinant anti-Ebola glycoprotein (GP) mAb 4G7 is a challenging antibody to manufacture in vitro; however, it is feasible through modified cell lines [103]. However, the original version of DMAb-4G7 had no expression in vivo as a human IgG1 pDNA-mAb. Patel et al. introduced single amino acid modifications into the Fab framework region to optimize DMAb-4G7 to restore in vivo expression [42]. Similar modifications were introduced into the framework of an anti-CTLA4 DMAb [100] and anti-HIV DMAbs [43] to achieve highly optimized in vivo expression [100]. Therefore, sequence optimizations have clearly played a critical role in in vivo expression of synthetic pDNA-mAbs. Further studies evaluating the contribution of amino acid modifications at the cellular level will provide highly valuable insights into the biology of in vivo-delivered antibodies. Like traditional antibody studies, approaches that reduce the immunogenicity of amino acid substitutions will also be valuable. Taken together, these modifications introduce a new approach to selectively modify pDNA-mAbs that would otherwise be discarded by traditional sequence liability screening methods, opening new avenues for delivery of highly potent and important antibodies.

Additionally, many of the Fc modifications identified in traditional antibody research can be applied to synthetic pDNA-mAbs. Introduction of the L234A, L235A (LALA) mutation to the pDNA-mAb Fc can abrogate Fc receptor (FcR) interactions, similarly to recombinant antibody [38, 101]. Other strategies to increase the duration of biologic antibodies in vivo include modifications in the Fc region that will increase interactions with the neonatal Fc receptor (FcRn) [104]. These modifications include M252Y/S254T/T256E (YTE) [105] in the Fc CH2 regions and M428L/N434S (LS) [106] and N434H [107] in the CH3 region. New modifications including YTW/KF [108] have been identified, and further study to determine potential benefits for nucleic acid-encoded mAbs will be highly informative to further extend expression in vivo. Overall, sequence engineering has been key to the success of synthetic DMAbs, and although similar modifications have not yet been studied for other pDNA-mAbs or mRNA-mAbs, such modifications will likely be important for diverse gene-encoded platforms.

### Studying Human IgG pDNA-mAbs in Animal Models

pDNA-mAb studies with species-matched Fc, such as studies with fully mouse (Fab and Fc) antibodies [36, 109] or fully sheep antibodies (Fab and Fc) [40], show long antibody expression in circulation. Not surprisingly, human antibody evaluation is challenging in animal models, as they develop a rapid immune response against foreign human Fc. It is also well-described that fully human antibodies have the potential to develop ADA responses in people. These immune responses can also be directed against the complementarity determining regions (CDRs) [110]. Additional approaches to assess fully human biologics in non-clinical and preclinical models are therefore needed. Patel et al. showed that the anti-DMAb antibody response is MHC class II-dependent and can be overcome in mouse models using T-cell depletion [39]. The T-cell-depleted animal is an important model, as a fully functional immune system returns in 14–21 days by normal thymopoiesis, allowing for complex immune and challenge studies to be performed. Other pDNA-mAb and mRNA-mAb studies utilized immunodeficient animal models such as athymic nude mice and RAG1 knockout, SCID, or NSG mice as alternative models to evaluate human IgG antibody expression and functionality. However, immune-deficient mice are unable to model the intricacies of a functional immune system and should be considered carefully as preclinical models. A recent study observed that NSG mice cleared recombinant mAb faster than other mouse models, presenting a significant challenge when evaluating biologics in this model [111]. Additional studies to help evaluate fully human pDNA-mAbs in mouse models would be highly informative for both non-viral and viral delivery and provide an important path forward for preclinical evaluation of in vivo-delivered antibodies [109].

## Cancer Immunotherapy

The first clinically relevant mAb approved by the FDA was orthoclone OKT3, an anti-CD3 antibody targeting T cells, in 1985, as a prophylaxis for organ transplant patients [112]. As the field evolved, the steady progression towards harnessing and arming the immune system to combat transformed cells intensified, leading to the discovery of rituximab, an anti-CD20 B-cell receptor antibody and the first successful mAb therapy approved for the treatment of hematological B-cell lymphoma [113].

Nivolumab (anti-PD1) and ipilimumab (anti-CTLA4) are two of the most successful checkpoint inhibitors currently on the market and have clinically been shown to increase overall survival and tumor progression for inoperable metastatic melanoma. According to the first-quarter average sales prices for 2019 from the Centers for Medicare and Medicaid Services (CMS), the mg/kg price for nivolumab and ipilimulmab was US$28 and US$153, respectively [114]. For an 80-kg, stage III or higher melanoma patient, a combination regimen of nivolumab and ipilimumab prescribes a 60- and 90-min IV injection every 3 weeks for four cycles, followed by nivolumab every 2 weeks until the patient presents a good progress report or can no longer tolerate treatment. This combination treatment alone amounts to about US$300,000 a year in total overall treatment, not accounting for other point-of-care measure costs such as post-infusion monitoring, which places a significant financial burden on the patient and their family as well as the overall healthcare system [115]. Genetic platforms such as pDNA-mAbs have the potential to address the significant challenge of cost and delivery time, without compromising safety and efficacy.

Muthumani et al. were the first to demonstrate a novel DMAb approach to target cancer, developing an anti-prostate-specific membrane antigen (anti-PSMA) encoded DNA in a mouse prostate cancer model [116]. In vivo, their study showed expression for this DMAb delivery of over 105 days post-injection, with important anti-tumor functionality, as tumor-challenged mice exhibited tumor regression and greater than 80% survival 58 days post-challenge compared to 0% survival in the vector control mice. The study also highlighted the potential for DMAb inducing antibody-dependent cellular cytotoxicity (ADCC) and phagocytosis (ADCP) with the recruitment of other immune cells such as natural killer (NK) cells and macrophages in identifying and clearing antigen-specific tumors. NK-cell-depleted mice injected with anti-PSMA DMAb and challenged were significantly less able to clear tumor and had less than 10% viability after a 56-day challenge compared to NK-expressing mice. Although in early stages of development, this treatment has the potential to offer an attractive clinical alternative/adjuvant therapy that can be coupled with current standard-of-care treatment options.

pDNA-mAbs have demonstrated that they can not only target and shrink specific tumors and those in hematological cancer models in vivo, but they can have a survival advantage and are being generated from a likely cost-effective platform. Duperret et al. reported on in vivo intermuscular DMAb delivery of mouse anti-CTLA4, and showed serum concentrations of up to 85 μg/mL, with a detectable level of expression of 15 μg/mL for over a year [100]. Mice challenged with tumors and subsequently administered anti-CTLA4 DMAb showed clearance in eight out of ten animals, with all demonstrating immunological memory after re-challenge by clearing 100% of the tumor. Investigators next designed and studied the functionality of DMAb-encoded anti-human CTLA4 ipilimumab and tremelimumab in vitro using donor-derived peripheral blood mononuclear cells (PBMCs) co-incubated with two different luciferase-expressing lymphoblast cancer models and showed a dose-dependent blockage of CTLA4, leading to increase luciferase expression. Perales-Puchalt et al. extended this work by engineering another important immune checkpoint inhibitor, anti-PD1, and showed expression and functionality supporting the possibility of administering both anti-CTLA4 and PD1 checkpoint as combination therapy [102]. A similar approach is currently being evaluated in the clinic with recombinant nivolumab and ipilimumab and is demonstrating greater efficacy in combination, prolonging overall survival in melanoma patients [117]. Therefore, pDNA-mAb delivery of checkpoint inhibitors is important, and the data from early mouse studies are supportive for continued development of high cancer value targets. Such developments provide opportunities to broaden the populations that receive such high-cost therapies.

### Cancer: Outlook

As the pDNA-mAb cancer approach continues through preclinical studies, new complementary applications are also being investigated. BiTEs are another parallel approach that is demonstrating promise in the clinic for bringing T cells into proximity with tumor surface antigens. Blinatumomab is the first BiTE antibody approved by the FDA. It targets hematological acute lymphoblastic leukemia by binding to both CD3 T-cell coreceptor and the malignant B-cell CD19 antigen. This antibody allows for contact-dependent activation of T cells and initiation of cytotoxicity against lymphoblastic cells. Although blinatumomab is highly promising in people, the BiTE has a short half-life of approximately 2 h. The current regimen requires IV delivery for 28 consecutive days and costs about US$90,000 per 28-day course of treatment for patients 45 kg and above [114, 118]. Therefore, a plasmid-encoded delivered BiTE would undoubtedly improve upon the relative short expression time length and could significantly lower the cost of such an important treatment. Building on previous DMAb studies, Perales-Puchalt et al. engineered a DNA-encoded bispecific T-cell engager (DBTE) that binds to CD3 T cells and the Her2 tumor target [119]. This study demonstrated consistent in vivo expression and cytotoxic activity for approximately 4 months, with potent functional activity showing 8/10 tumor regression/clearance in mice > 40 days after tumor challenge.

Stadler et al. encoded a similar bispecific antibody in mRNA, encoding the tumor-associated antigen claudin, a tight junction protein and epithelial cell adhesion molecule (EpCAM), which is overexpressed in colorectal, prostate, ovarian, and endometrial cancers [120]. They detected the translated mRNA bispecific antibody, with CD3 and claudin or EpCAM-binding capability, in as little as 6 h following intravenous delivery; plasma at the 6-h time point showed in vitro cytotoxicity at above 90%, with expression still present at the 50% level through day 6 post-injection of mRNA. In vivo data demonstrated three mRNA doses of 3 µg/mouse by IV injection compared to ten doses of 200 µg/kg of recombinant CD3 and claudin protein had comparable median tumor volume. The mRNA-encoded bispecific antibody showed earlier and rapid tumor regression, with fewer overall doses. Overall, both mRNA-encoded bispecific antibodies and DBTE pDNA-mAb approaches offer the ability to harness T-cell functionality in blocking tumor antigen function and initiating potent cytotoxicity, but vary in length of expression, stability, and dosing regimen, with DNA having longer and more durable expression from a single injection compared to mRNA. These are interesting and promising and likely complementary strategies for cancer immunotherapy that deserve significant attention.

## Infectious Disease Control

There is tremendous potential for mAb delivery to have an impact in the infectious diseases arena. Through advancements in antibody discovery technologies, highly specific mAbs are being isolated directly from convalescent humans with activity against anti-microbial resistant bacteria, emerging and re-emerging viral pathogens, and parasitic and fungal diseases. Yet, palivizumab (Synagis), an anti-respiratory syncytical virus mAb, is the only commercially successful mAb approved as a standalone intervention to prevent an infectious disease. Synagis costs can be > US$12,000 for a five-dose (15 mg/kg/dose) regimen, administered at 1-month intervals [77]. Bezlotoxumab (Zinplava), an antibody targeting treatment of *Clostridium difficile*, was approved in 2016 and is indicated to reduce recurrence of *C. difficile* infection (CDI) in patients 18 years of age or older who are receiving antibacterial drug treatment of CDI and are at a high risk for recurrence. According to the CMS, Zinplava currently costs > US$4500/g [114] and has a recommended dose of 10 mg/kg IV over 60 min [114]. Two additional mAbs, raxibacumab and obiltoxaximab, have been approved for treatment of inhalational anthrax via the FDA Animal Efficacy Rule. The Animal Rule serves as a mechanism to gain approval for drugs and biologics using animal data in situations where it is not feasible or ethical to perform human studies. This mechanism is therefore reserved for unique circumstances such as evaluation of countermeasures against biological select agents. Although many antibodies are currently under preclinical and clinical trial evaluation, in vivo mAb potency, dosage, delivery, and patient cost remain important obstacles that impede administration for the treatment of infectious diseases. Current antibody limitations impede widespread delivery for seasonal outbreaks and epidemics for influenza viruses. Repeat doses are required to lower HIV viral loads. The development and manufacturing timeline for rapid response to emerging infectious disease outbreaks, for example, Ebolavirus, is long and requires cold-chain storage, which may limit deployment in resource-limited areas. Therefore, in vivo nucleic acid-encoded antibody delivery represents an attractive approach for antibody administration targeting infectious diseases.

### Viral Infections and Speed to Intervention

**Fig. 5:**
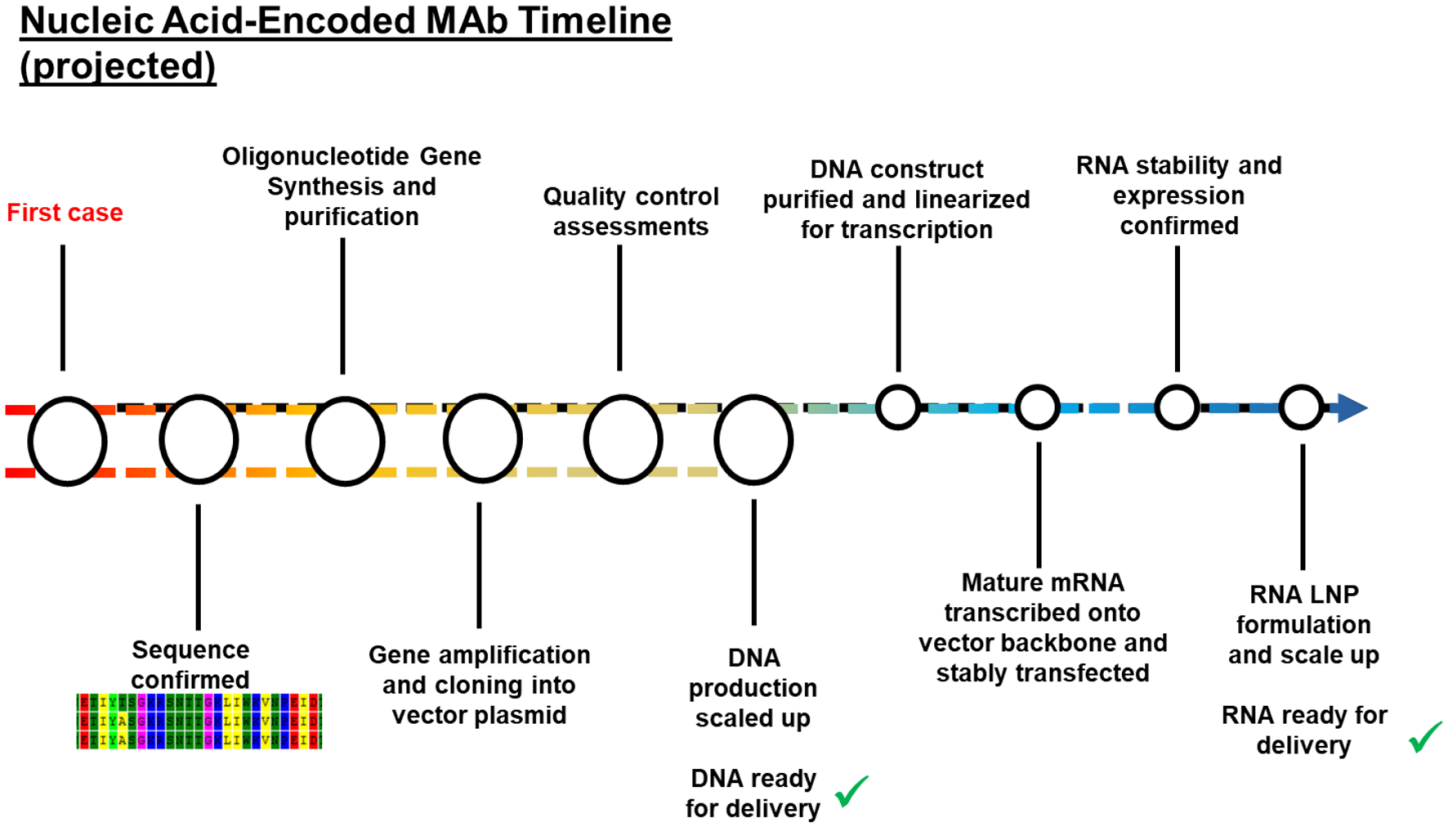
Nucleic acid-encoded mAb timeline. Antibodies are identified from infected individuals and sequences are confirmed to binding and/or neutralize the target antigen. The mAb gene is then synthesized and cloned into a DNA plasmid, followed by amplification and DNA scale up. At this point, all pDNA-mAbs are ready for in vivo administration. mRNA requires additional processing, including DNA linearization, followed by mRNA transcription. RNA stability must be evaluated and formulated into LNPs before an mRNA-mAb is ready for in vivo delivery. *LNP* lipid nanoparticle, *mAb* monoclonal antibody, *mRNA* messenger RNA, *mRNA-mAb* mRNA-encoded mAb, *pDNA-mAb* plasmid DNA-encoded mAb

Both DNA- and mRNA-mAbs represent potential rapid delivery platforms for emerging infectious disease control. Both platforms start with initial antigen-specific mAb sequence identification. For pDNA-mAbs, antibody sequences are nucleotide and amino acid optimized using in silico methods, followed by rapid gene synthesis and insertion into the DNA vector backbone. pDNA-mAb DNA can then be amplified in small or large batches, and is ready for in vivo delivery within a short timeline (Fig. 5). The process for mRNA-mAb development is similar, but requires additional manufacturing steps. Antibody sequences are engineered into a DNA plasmid that includes a 5′UTR, 3′UTR, and polyadenylation signal. This is followed by plasmid DNA amplification in bacteria. mRNA-mAbs must then be transcribed from the DNA and formulated and then re-purified and stored for administration in an LNP. This last step is critical for mRNA stability and extends the timeline for delivery in vivo.

Several studies have demonstrated the protective efficacy in mice for pDNA-mAbs and mRNA-mAbs targeting dengue virus [101], influenza A and B viruses [36, 37, 97], Ebolavirus [36, 42], Zika virus [38], CHIKV [121], rabies [15], and HIV [49].

#### Anti-Dengue Virus pDNA-mAbs

Close to 4 billion people are estimated to be at risk for dengue virus infection worldwide, mostly in developing countries and in resource-poor settings [122]. Flingai et al. demonstrated the first generation of DMAb engineering and delivery of anti-dengue virus antibodies [101]. They evaluated both a wild-type (wt) and variant DMAb incorporating the LALA mutation into the Fc region to mitigate dengue secondary infection. The anti-dengue virus DMAb achieved peak expression levels of around 1 μg/mL for at least 19 weeks in mice and demonstrated neutralization of dengue virus serotypes 1–3. Both the anti-dengue virus wt-DMAb and LALA-DMAb demonstrated protection against primary dengue infection. The LALA-DMAb demonstrated protection against dengue virus primary infection, as well as protection in a mouse model of antibody-dependent enhancement that models dengue secondary infection. Overall, this demonstrates the portability of traditional mAb engineering strategies to pDNA-mAb platforms for unique strategies against emerging viral infections.

#### Anti-influenza Virus pDNA-mAbs

An estimated 45 million people in the USA were infected by influenza viruses during the 2017–2018 influenza season, resulting in 810,000 hospitalizations and 61,100 deaths [123]. Advancing mAb delivery could have an important impact on lowering overall influenza disease burden during seasonal influenza epidemics and against potential pandemic viruses. Yamazaki et al. were one of the first to show in vivo expression of a pDNA-mAb anti-influenza A virus hemagglutinin (HA) mouse IgG1 antibody [97]. They demonstrated initial expression of approximately 1 μg/mL anti-HA antibody in mice. Utilizing an early generation electroporation (EP) device, they showed the benefit of delivery and hyaluronidase pre-treatment of the muscle to increase muscle uptake of DNA, resulting in serum levels of > 15 μg/mL in mice and expression for 50 days. The pDNA-mAb expressed anti-HA antibody was detectable in lung bronchioalveolar lavage and protected mice against lethal A/H1N1/Puerto Rico/8 virus challenge. Following these studies, Elliott et al. engineered human IgG1 DMAbs targeting pan-influenza A virus and pan-influenza B virus HA proteins (A/HA and B/HA, respectively) [37]. Using an advanced EP device and hyaluronidase pre-treatment, the anti-HA DMAbs were expressed to levels of > 10 μg/mL against A/HA and > 30 μg/mL against B/HA, with expression for 70 days. Dose titration of both A/HA and B/HA DMAbs was performed and evaluated in parallel with recombinant antibody to demonstrate equivalency to protein antibody. The anti-A/HA DMAbs demonstrated protection against seasonal H1 and H3 viruses, as well as neutralization of several group 1 and group 2 subtypes. Similarly, anti-B/HA DMAbs protected against both B/Yamagata and B/Victoria lineage virus infection in mice. Additionally, a combination of both anti-A/HA and anti-B/HA DMAbs protected against both A/California/7/2009 and B/Florida/4/2006 virus challenges, demonstrating the potential for co-delivery, and protected against homologous re-challenge. In a parallel study, Andrews et al. monitored the delivery of anti-influenza A/H1/HA or A/H3/HA mouse IgG2a pDNA-mAbs long term in mice [36], observing similar pharmacokinetics as Elliot et al., supporting the data generated with human IgG1 DMAbs in mice. They reported a maximum expression with one A/H3/HA pDNA-mAb of 5 μg/mL in BALB/c mice, with expression lasting for 44 weeks. A combination of all three A/HA pDNA-mAbs protected mice against lethal A/H1N1/WSN/33 and A/H3N2/Aichi/2/68 challenges and protected against virus re-challenge. Taken together, these three studies highlight mouse IgG pDNA-mAbs as in vivo antibody development tools and the potential to evaluate fully human IgG1 pDNA-mAbs in the mouse model to demonstrate preclinical protection against seasonal and pandemic influenza A and B viruses.

#### Anti-ebolavirus pDNA-mAbs

The 2013–2016 Ebolavirus disease (EVD) epidemic was the largest to date, with over 28,000 confirmed cases and over 11,000 deaths. There is an ongoing outbreak in the Democratic Republic of Congo, and additional gene-encoded alternatives could be useful for EVD infection control to support post-exposure vaccines and therapeutics. Antibodies against the *Zaire ebolavirus* GP are demonstrating promising therapeutic protection against EVD. The three-mAb ZMapp cocktail was the first antibody combination to demonstrate promise in an EVD outbreak setting [124]. Andrews et al. encoded the ZMapp cocktail antibodies into their pDNA-mAb platform [36], showing delivery of each individual mouse IgG2a antibody to a maximum of 10 μg/mL and 30 μg/mL for combined delivery and 13 weeks of in vivo expression. The pDNA-ZMapp was delivered to mice, followed by challenge 28 days later with mouse-adapted Ebolavirus, demonstrating protection against lethal infection. In a separate study, Patel et al. engineered 26 different anti-GP DMAbs, three mouse-human chimeras, and 23 fully human IgG1 DMAbs [42]. They performed a series of sequence, formulation, and delivery optimizations to achieve peak expression of DMAb-11 targeting the GP fusion loop of > 50 μg/mL and > 26 μg/mL for DMAb-34, expressing for 365 days following administration. They compared anti-GP DMAb delivery in parallel with recombinant antibody. These studies demonstrated functional equivalency to bind antigen and that DMAbs bind the same molecular epitope as the parent recombinant mAb. Both anti-GP DMAb-11 and DMAb-34 protected mice against lethal mouse-adapted Ebolavirus challenge as single candidates as well as when delivered together. These studies also showed that anti-Ebolavirus GP DMAbs are protective in mice when delivered only 8 days before lethal challenge and also provide long-term partial protection when challenged 82 days post-administration. These studies are the first demonstration of the potential for prophylactic delivery of gene-encoded mAbs to prevent Ebolavirus infection.

#### Anti-Zika Virus pDNA-mAbs

Over 2 billion people are at risk for Zika virus infection [125]. Building on the early studies with the anti-dengue DMAb, Esquivel et al. engineered anti-Zika virus wt-DMAb and LALA-DMAb targeting the virus E protein [38]. They showed average expression of 27 μg/mL for the wt-DMAb and 62 μg/mL for the LALA-DMAb, with 70 days of expression. Both anti-Zika virus wt-DMAb and LALA-DMAb protected AG129 mice against Zika virus challenge and, in addition, protected against Zika-related damage to the testes. The investigators next delivered the anti-Zika wt-DMAb to rhesus macaques using a sequential administration strategy on days − 10, − 7, and − 4 before challenge with Zika virus PRVABC59 on day 0. Macaques expressed on average 1 μg/mL of wt-DMAb at the time of challenge, and four out of five animals controlled Zika virus infection. This was the first demonstration that a nucleic acid-encoded antibody can control an infectious disease challenge in a larger animal model, further supporting translation of the DMAb platform for administration in people. This Zika virus DMAb candidate is now in a human trial (NCT03831503), which we will discuss in more detail later in this review (Sect. 6).

#### Anti-HIV pDNA-mAbs

There are approximately 37 million people living with HIV, with the envelope GP (Env) being the primary target for immune responses. Due to the high diversity of Env proteins, vaccine development and therapy have been challenging. Several Env-targeted mAbs have been identified and shown to broadly neutralize multiple circulating HIV-1 strains (reviewed in Ref. [126]). Wise et al. engineered a panel of 16 broadly neutralizing HIV-1 antibodies as DMAbs and delivered them in mice and non-human primates [43]. The DMAbs, delivered alone or in combination, demonstrated long expression for 300 days and neutralized against the global panel viruses in an in vitro neutralization assay. The data highlight how the combination of sequence optimizations, formulation, delivery, and animal model development can lead to a significant in vivo expression level using pDNA-mAb platforms.

#### mRNA-mAbs Targeting Infectious Diseases

To date, mRNA-mAbs, formulated in LNP, have been evaluated targeting rabies [15], HIV [49], and CHIKV [127]. Human anti-rabies immunoglobulin and the rabies vaccine must be delivered immediately following potential rabies exposure. Thran et al. evaluated an mRNA-mAb platform for rapid expression of an anti-rabies antibody. They observed peak human IgG titers of 10 μg/mL within 4–6 h post-administration and expression for 8 days, before it declined [15]. This anti-rabies mRNA-mAb provided both pre-exposure (day − 1) and post-exposure (2 h post-infection) protection against lethal rabies virus challenge in mice. In this study, an anti-influenza B mRNA-mAb was also described as a control, expressing at similar levels to the anti-rabies mRNA-mAb.

Several promising broadly neutralizing antibodies have been identified targeting the HIV env protein and are demonstrating promising results for HIV treatment (reviewed in Ref. [128]). Pardi et al. [49] encoded the HIV-VRC01 antibody into a nucleoside modified mRNA. They achieved peak expression levels of > 150 μg/mL 1 day post-administration, which lasted for 11 days. Eight weeks of expression were achieved with weekly mRNA-VRC01 delivery for 4 weeks. Protection against SF162 and JR-CSF HIV-1 challenges was observed in humanized mice receiving the mRNA-VRC01, similar to recombinant mAb.

Recently, Kose et al. described an anti-CHIKV mRNA-mAb that was delivered by IV injection to AG129 mice that were then challenged with CHIKV. They demonstrated dose-dependent protection against the virus, with no viral load and protection against arthritis and muscoskeletal disease in competent C57BL6 mice [127]. They further evaluated expression in cynomolgus macaques, indicating rapid expression of mRNA, and repeat dosing at a 7-day interval. However, the infusion duration was over 60 min, which a significant time for field delivery. Additional studies to reduce mRNA-mAb infusion times would greatly improve the platform to make it more accessible in low-resource settings.

### Bacterial Infections

Antibodies are promising for control of antimicrobial resistant organisms and other serious bacterial infections. Each year, at least two million people acquire antimicrobial resistant infections, including multi-drug resistant *Pseudomonas aeruginosa*. First-line antimicrobials are rapidly becoming ineffective, and alternative biologics are urgently needed. Patel et al. described the first delivery of an engineered, non-natural bispecific immunoglobulin using the DMAb platform [39], achieving expression levels of 8 μg/mL for 100 days. This DMAb targeted two *P. aeruginosa* proteins (PcrV type III secretion system and Psl exopolysaccharide), protecting against lethal pneumonia in a mouse challenge model. Lower levels of pro-inflammatory cytokines, organ colonization, and lung pathology were observed following both monospecific and bispecific DMAb delivery, similar to the recombinant mAb positive control. Additionally, the bispecific DMAb demonstrated adjunctive activity when delivered in conjunction with a carbapenem-family antibiotic. This demonstrates the potential for DMAb delivery as a standalone intervention or alongside first-line antimicrobials. New-generation pDNA-mAb development to protect against ESKAPE (***E****nterococcus faecium*, ***S****taphylococcus aureus*, ***K****lebsiella pneumoniae*, ***A****cinetobacter baumannii*, ***P****seudomonas aeruginosa*, ***E****nterobacter species*) pathogens and other resistant bacteria have the potential to dramatically impact infection control and the spread of anti-microbial resistance in both hospital and community settings.

Around 30,000 cases of Lyme disease are reported each year to the US Centers for Disease Control (CDC); however, it is likely that the actual number of cases each year is closer to 300,000 in the USA [129]. Wang et al. engineered an anti-*Borrelia burgdorferi* DMAb candidate expressing a transmission-blocking antibody targeting OspA [130]. Initial expression of the wt-DMAb averaged 5.7 μg/mL, and sequence optimizations further increased expression to 6.7 μg/mL. The modified DMAb retained bactericidal activity comparable to the original DMAb and recombinant parent mAb. Both the wt-DMAb and modified DMAb were protective against *B. burgdorferi* challenge in mice. This supports the potential for a DMAb approach to protect against Lyme disease transmission from tick to host.

To date, the only mRNA-mAb targeting a bacterial pathogen is against botulinum toxin A1 [15]. Botulism is caused by the toxin from *Clostridium botulinum* and requires immediate delivery of anti-toxin to prevent paralysis. Thran et al. encoded a camelid VHH antibody targeting botulinum neurotoxin serotype A (BoNT/A), with levels reaching > 100 μg/mL in less than 24 h post-administration. The mRNA-mAb successfully protected against BoNT/A toxin challenge when delivered 6 h post-exposure. Additional development of mRNA-mAbs for rapid delivery against bacterial toxins that require protection within hours of exposure has the potential to be important.

### Infectious Diseases: Outlook

**Fig. 6:**
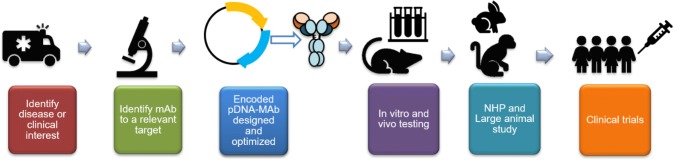
pDNA-mAb pipeline. *NHP* non-human primate, *pDNA-mAb* plasmid DNA-encoded mAb

pDNA-mAb platforms can be utilized to rapidly evaluate potential mAb candidates. Antibodies isolated from infected or convalescent individuals can be directly engineered into pDNA-mAb for in vivo evaluation in a biologically relevant environment. The top candidates from such a pipeline can then be rapidly tested in animal models. The simplicity and flexibility of synthetic DNA plasmid enables rapid scaling for administration in a person. This could open a new paradigm for rapid antibody administration in response to an outbreak (Fig. 6).

Several funding agencies have expressed interest in this process for the rapid development of nucleic acid biologics, including the National Institutes of Health, Defense Advanced Research Projects Agency (DARPA), Biomedical Advanced Research and Development Authority (BARDA), and the Bill and Melinda Gates Foundation (BMGF). The DARPA Pandemic Prevention Program (P3) was initiated in 2017, with the goal of pandemic prevention in less than 60 days through rapid deployment of nucleic acid-vectored synthetic DNA or mRNA technologies delivering protective antibody. Similar to what is shown in Figs. 5 and 6, the program is supporting rapid isolation, nucleic acid development, and delivery approaches to achieve protective in vivo expression levels within 3 days of administration. In the final stage of the DARPA P3 program, the DNA and mRNA technologies will be tested in a capability test with an unknown pathogen.

Importantly, in an earlier study, Muthumani et al. delivered an anti-CHIKV DMAb in combination with a CHIKV DNA vaccine, demonstrating both immediate and persistent protection against lethal challenge in mice [121]. This study supports the concept that pDNA-mAbs have the potential to be delivered in conjunction with protective vaccination, providing rapid protection, with the pDNA-mAb to cover the period needed to establish vaccine-induced immunity.

The concept of immediate and persistent protection introduced by Muthumani et al. is intriguing. Since pDNA-mAbs express for months, they have the potential to afford a new way for both pre-exposure and post-exposure prevention that has previously been unachievable with recombinant biologics. Additional studies to confirm immunogenicity and protective efficacy with combined pDNA-mAb-vaccine delivery would be informative.

By comparison, there are much fewer studies with mRNA-mAb delivery against infectious diseases. As the field advances, it will be important to utilize the information coming out of these studies to advance new approaches and to better define the pathway for development.

## Translation to Humans

**Fig. 7:**
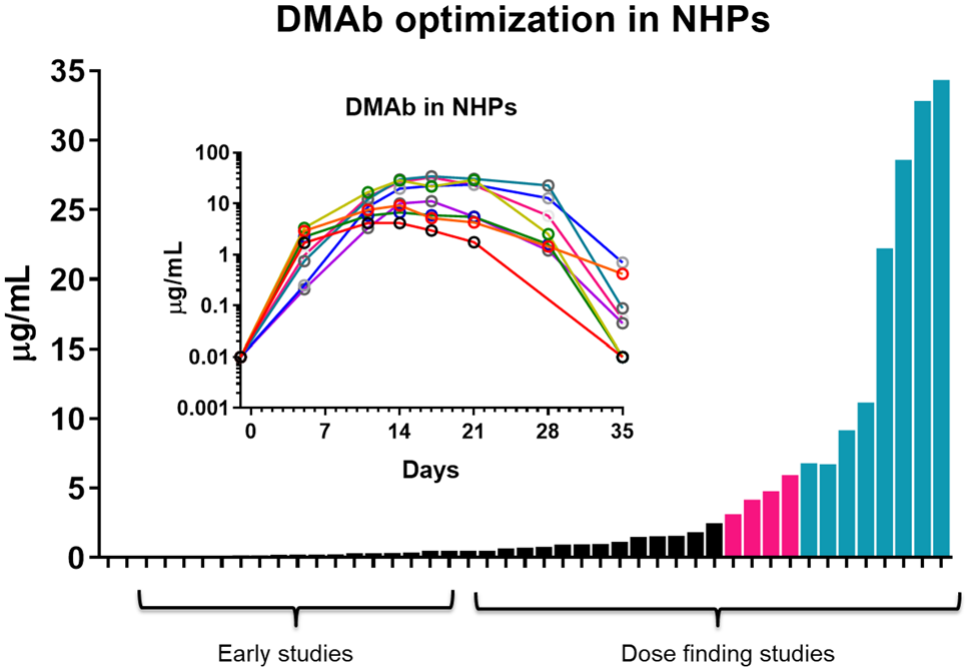
DMAb expression NHPs following sequence, delivery, and formulation optimizations. *DMAb* DNA-encoded mAb, *NHP* non-human primate. Black = early and low-expression studies. Magenta = dose-finding studies leading to significant increases in expression. Teal = dose-finding studies using optimized formulation and delivery (individual teal group animals are show in the inset)

Large animal studies and data from human clinical trials will be highly informative for gene-encoded mAb delivery platforms. An older study by Tjelle et al. demonstrated in vivo expression of a mouse IgG2a pDNA-mAb in sheep, observing expression levels as high as 50 ng/mL before development of sheep anti-mouse antibodies [94]. More recently, Hollevoet et al. describe delivery of fully sheep antibodies, achieving expression levels up to 3.5 µg/mL [40]. This is the first study to demonstrate microgram expression levels in larger animals more similar in mass to people. Previously, Timmerman et al. evaluated plasmid DNA delivery of an anti-idiotype antibody targeting B-cell lymphoma, observing modest anti-tumor activity [131]. However, this study did not measure in vivo expression levels. Building on these previous studies, significant advancements in formulation and delivery are now leading to higher expression levels in monkeys (Fig. 7, not previously published). At the start of these studies, animals received four to six IM injections (animals in black); however, only 0–30 ng/mL human IgG expression levels were detectable in rhesus macaques following DMAb delivery. Following a series of optimizations, expression increased to a 4–7 µg/mL (summary of data from non-human primates receiving 2–9 mg total DNA in 4–6 injection sites) (Fig. 7, magenta), and additional device/delivery optimizations, including hyaluronidase treatment, have led to further increases, now to levels > 30 µg/mL (6 mg total DNA injection, six injections), which express for 35 days before elimination via the development of macaque anti-human antibodies (Fig. 7, teal). Wise et al. demonstrated that using an optimized delivery strategy with a 6-mg dose, divided across six injection sites, anti-HIV DMAb delivery can achieve in vivo expression levels as high as 34.4 µg/mL [43]. These expression levels parallel mRNA expression levels, which are at > 30 μg/mL in rhesus macaques [127]. Only AAV can surpass these levels in macaques; however, virus take in animals is reported to be not consistent with AAV, and there were wide variations in delivery observed, ranging from 0 to 270 µg/mL across multiple studies.

Dose scaling is an important consideration for non-viral and viral vector-encoded antibodies. Plasmid DNA is transiently transfected into muscle and may persist in the tissue for a few weeks. Likewise, viral vectors typically persist in tissues for a few months, or permanently if integrated. This is quite different to a biologic, for which the maximum amount of drug is at the time of infusion. mRNA is most similar to traditional protein biologics, as it is rapidly expressed and degraded. Therefore, initial empiric dose escalation studies will be important to set the bar and move the field to optimal doses in the clinic. Additional optimizations including sequential administration [38], formulation, and delivery technology are rapidly developing and will likely contribute to increases in delivery in vivo.

Recently, Priddy et al. evaluated in vivo administration of an AAV-vector encoding the anti-HIV PG9 (AAV-PG9) antibody in people [132]. No expression of PG9 was detected in participants’ sera, although PG9 was detected in muscle biopsies. One possibility for the lack of detection is the high enzyme-linked immunosorbent assay (ELISA) limit of detection (2.5 µg/mL), and more sensitive assays are likely needed as development continues. Furthermore, ADA was observed against both the AAV vector and PG9, suggesting that it did induce an immune response. More studies are needed in this regard to build on this initial study.

A first-in-human phase I trial evaluating the safety, tolerability, and pharmacokinetic expression of the anti-E protein Zika virus DMAb (INO-A002) is currently underway (NCT03831503; trial start date: February 7, 2019; estimated study completion: February 2021). In this study, dose escalation starting at 0.5 mg up to 4 mg of INO-A002 is being evaluated in healthy human volunteers. Supported by the preclinical data in mice and macaques, the human study will deliver DMAb in formulation with recombinant human hyaluronidase to improve drug dispersion. In parallel, a first-in-human mRNA-mAb phase I trial evaluating the safety, tolerability, pharmacokinetics, and pharmacodynamics of an mRNA-encoded anti-CHIKV antibody (mRNA-1944) is also underway led by Moderna (NCT03829384; trial start date: January 22, 2019; estimated study completion: September 2020). This study will evaluate dose escalations starting with 0.1 up to 1 mg/kg via IV administration. At a recent presentation at the Oligonucleotide Therapy Society (2019), Moderna presented mRNA-1944 data demonstrating expression in people at levels > 1 µg/mL even in the lowest dose group [53]. Following IV infusion with mRNA-1944, peak expression was reached within 24 h, declining afterwards, with detectable levels for at least 84 days, with the 0.3 mg/kg dose. Patients were treated with anti-histamine, and at 0.6-mg/kg doses there were multiple adverse events observed. However, this trial is a very positive development for nucleic acid biologics. While much more work is needed, this study is an important advance for the field. Together, these trials will be informative to help understand additional optimizations that must be undertaken to improve human translation of nucleic acid-encoded antibodies and will be significant for the field.

## Looking Forward

The clinical use of mAbs has had a dramatic impact on improving human health and affording a better quality of life for many individuals. However, use has been restricted to disease populations with high commercial value, due in part to the high cost of development of these biologics. The future for pDNA-mAbs and gene-encoded antibodies is promising and presents interesting opportunities to make life-saving biologics more accessible. pDNA-mAbs build on existing antibody technologies and can be engineered to deliver new types of biologics, such as bispecifics [39], trispecifics, and alternative forms and isotypes. Other modifications including half-life extension to improve interactions with the neonatal FcRs and glycosylation profiles would be highly informative for modulation of in vivo duration and effector functions. Further in vivo modification may be accomplished through co-delivery of different enzymes to direct post-translational processing. pDNA-mAbs and mRNA are opening a new direction in the field that was previously limited to biologics, with powerful potential applications for in vivo delivery, including new classes of non-immunoglobulin biologics such as T-cell and other cell engagers, as part of this new approach for advancing a novel area of “nucleic acid-based medicines.” The initial studies described in this review represent the tip of the iceberg for their potential applications, as we are just entering the clinic. While early, these highly disruptive nucleic acid technologies have the potential to transform our approaches to the development of biologics, opening up new areas of opportunity for disease treatments of global importance for patients and for animal health.
