# Correction to: In Vivo Delivery of Nucleic Acid-Encoded Monoclonal Antibodies

**DOI:** 10.1007/s40259-020-00424-z

**Published:** 2020-04-02

**Authors:** Ami Patel, Mamadou A. Bah, David B. Weiner

**Affiliations:** Vaccine and Immunotherapy Center, The Wistar Institute, 3601 Spruce Street, Philadelphia, PA 19104 USA

## Correction to: BioDrugs 10.1007/s40259-020-00412-3

The article In Vivo Delivery of Nucleic Acid-Encoded Monoclonal Antibodies, written by Ami Patel, Mamadou A. Bah and David B. Weiner, was originally published under a CC BY-NC 4.0 license, but has now been made available under a CC BY 4.0 license. The article is forthwith distributed under a Creative Commons Attribution 4.0 International License (https://creativecommons.org/licenses/by/4.0/), which permits use, sharing, adaptation, distribution and reproduction in any medium or format, as long as you give appropriate credit to the original author(s) and the source, provide a link to the Creative Commons licence, and indicate if changes were made. The PDF and HTML versions of the paper have been modified accordingly.

The original article has been corrected.

**Open Access** This article is licensed under a Creative Commons Attribution 4.0 International License (https://creativecommons.org/licenses/by/4.0/), which permits use, sharing, adaptation, distribution and reproduction in any medium or format, as long as you give appropriate credit to the original author(s) and the source, provide a link to the Creative Commons licence, and indicate if changes were made.

